# Supplementary material for: Specific Neuroligin3–αNeurexin1 signaling regulates GABAergic synaptic function in mouse hippocampus
Source: eLife. 2020 Dec 23;9:e59545. doi: 10.7554/eLife.59545 (PMC7758064; doi:10.7554/eLife.59545)
Supplement: Source code 1. [file elife-59545-code1.zip › Rsourcecode.docx]

# counts to TPM conversion

# https://support.bioconductor.org/p/91218/

tpm <- function(counts,len) {

x <- counts/len

return(t(t(x)*1e6/colSums(x)))

}

# use Mus_musculus.GRCm38.GC_lengths.txt retrieved from BioMart

in_f1 <- "G709_G556.csv"

d1 <- read.csv(in_f1, header=TRUE, row.names=NULL)

d3 <- read.table("Mus_musculus.GRCm38.GC_lengths.txt", header=TRUE, row.names=NULL, sep="\t", quote="")

d3<-d3[,c(1,2)]

d2<-merge(d1,d3, by.x="name",by.y="ID")

d5<-tpm(d2[,c(2:20)],d2[,21])

rownames(d5)<-d2[,1]

write.csv(d5,"G709_G556_TPM.csv")
